# Mucosal microbiota and intraepithelial lymphocyte remodeling link maternal immune activation to neuroimmune outcomes in female and male offspring

**DOI:** 10.3389/fimmu.2026.1945751

**Published:** 2026-09-18

**Authors:** Ana Raquel Pereira-Santos, Soraia Jesus-Faria, Emanuel Candeias, Ana Maranha, Igor Tiago, Paulo Rodrigues-Santos, Nuno Empadinhas, Sandra Morais Cardoso

**Affiliations:** 1 Center for Neuroscience and Cell Biology (CNC), University of Coimbra, Coimbra, Portugal; 2 Centre for Innovative Biomedicine and Biotechnology (CIBB), University of Coimbra, Coimbra, Portugal; 3 Ph.D. Programme in Experimental Biology and Biomedicine (PDBEB), Institute for Interdisciplinary Research, University of Coimbra, Coimbra, Portugal; 4 Faculty of Science and Technology, University of Coimbra, Coimbra, Portugal; 5 Faculty of Medicine, University of Coimbra (FMUC), Coimbra, Portugal; 6 Center for Investigation in Environment, Genetics and Oncobiology, University of Coimbra, Coimbra, Portugal; 7 Clinical and Academic Centre of Coimbra (CACC), ULS of Coimbra, Coimbra, Portugal

**Keywords:** gut inflammation, intraepithelial lymphocytes, maternal immune activation, microglia, mucosa-associated microbiota, prefrontal cortex, schizophrenia

## Abstract

**Background:**

Maternal immune activation (MIA) is associated with increased risk for neurodevelopmental disorders, including schizophrenia and autism spectrum disorder, yet its long-term impact on intestinal immune-microbial programming remains incompletely understood. Here, we investigated whether lipopolysaccharide (LPS)–induced MIA drives persistent alterations in the offspring mucosal immune environment, with a focus on intraepithelial lymphocytes, mucosa-associated microbiota, systemic cytokines, and prefrontal cortical immune-related readouts.

**Methods:**

MIA was induced in wild-type BALB/cByJ mice via intraperitoneal LPS injection at gestational day 12.5. Male and female offspring were assessed at 10 weeks of age for gut, systemic, and prefrontal cortex (PFC) immune alterations.

**Results:**

MIA offspring displayed a regionally restricted intestinal immune signature, with more pronounced alterations in the ileum than in the colon. Ileal changes included increased inflammatory cytokine signaling and remodeling of IEL populations, particularly TCRγδ CD8αα cells, together with changes in TCRαβ IEL subsets. Analysis of the mucosa-associated microbiome revealed parallel changes in ileal microbial community structure, including alterations in segmented filamentous bacteria and *Lactobacillus*. These mucosal alterations co-occurred with increased plasma IL-6 in both female and male offspring, while plasma IL-17 was selectively increased in females. In the PFC, MIA was associated with cytokine changes and divergent microglial morphological profiles, suggesting that prenatal immune challenge produces neuroimmune alterations that may differ in sex, timing, magnitude, or cellular expression.

**Conclusions:**

Collectively, these findings identify the mucosa-associated microbiota-IEL interface as a previously underexplored component of MIA-induced immune remodeling. Our study provides an integrated framework in which persistent ileal immune and microbial alterations co-occur with systemic inflammation and cortical neuroimmune changes in MIA offspring, supporting future mechanistic studies of gut–brain immune communication in neurodevelopmental disorders.

## Graphical Abstract

**Figure:**
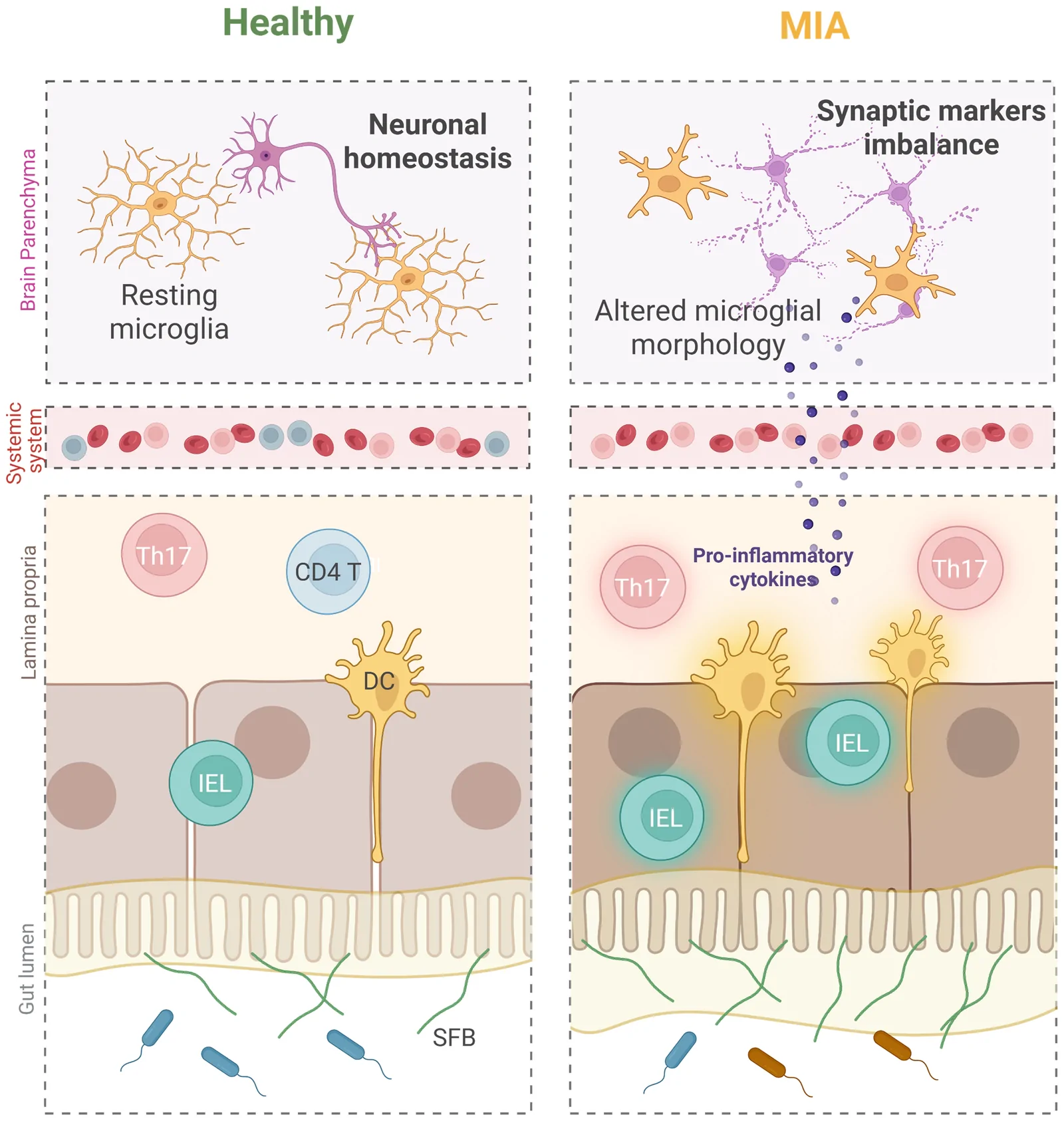
Gut immunity dysregulation in schizophrenia. In a maternal immune activation (MIA) model, prenatal immune perturbation drove to immune alterations, including elevated IL-17, epithelial stress, and altered intraepithelial lymphocyte (IEL) populations in the gut. These changes were linked to microglial dysfunction, altered microglial morphology, and neuroinflammation, contributing to cognitive and behavioral impairments. Together these findings highlight the microbiome-gut-brain axis as a key mediator of neuroimmune interactions in schizophrenia.

## 1 Introduction

Prenatal immune challenges, including maternal infection and immune activation during pregnancy, are increasingly recognized as early life environmental factors capable of shaping neurodevelopmental trajectories and later vulnerability to neuropsychiatric outcomes (1–3). Because schizophrenia (SCZ) is a heterogeneous neurodevelopmental psychiatric disorder that typically emerges during late adolescence or early adulthood (4–6), maternal immune activation (MIA) models are best viewed as experimental paradigms of neurodevelopmental risk rather than as reproductions of the full complexity of SCZ.

Accordingly, MIA paradigms are relevant to SCZ-related vulnerability, autism spectrum disorder, and other neurodevelopmental conditions, with behavioral outputs that may map onto shared dimensions such as altered social behavior, exploratory activity, sensorimotor gating, anxiety-like behavior, or cognitive flexibility rather than to one diagnostic entity alone (1–3, 6). Beyond the brain, increasing clinical evidence supports the relevance of the gut-immune-brain axis in SCZ. Patients with SCZ show alterations in gut microbiota composition and mucosal immune-related signatures, and these changes have been associated with systemic inflammatory markers, intestinal permeability markers, metabolic status, and clinical heterogeneity (7–9). These human data do not imply a single gut-derived cause of SCZ, but they support the idea that mucosal immunity and microbiota composition may contribute to biologically distinct subgroups within the broader SCZ spectrum.

Intraepithelial lymphocytes (IELs) are positioned at the interface between the intestinal epithelium, luminal microbes, and host immunity. They include conventional and unconventional TCRαβ and TCRγδ subsets that contribute to epithelial surveillance, tissue repair, and immune restraint but can also participate in inflammatory programs when epithelial stress or microbial cues are altered (10–12). The mucosa-associated microbiota is particularly relevant in this context because organisms that closely interact with the epithelium, such as segmented filamentous bacteria (SFB), can shape Th17/IL-17–associated immunity and IEL responses, whereas commensal *Lactobacillus* species are often associated with barrier-supportive and immunoregulatory effects (13–15).

Previous MIA studies have reported long-lasting changes in fecal microbiota composition and intestinal immune function in offspring, most often using C57BL/6 mice and poly(I:C)-based immune activation (16–18). LPS- and poly(I:C)-induced MIA models are not interchangeable, as they engage different innate immune pathways and can yield strain-, dose-, timing-, and sex-dependent outcomes (19, 20).

Here, we identify the mucosa-associated microbiota-IEL interface as a persistent and previously underexplored target of low-dose *E. coli*–derived LPS-induced MIA. Adult MIA offspring displayed ileum-predominant immune alterations, remodeling of IEL subsets, including TCRγδ CD8αα cells, and changes in mucosa-associated microbial communities, particularly taxa linked to mucosal immune regulation. These intestinal changes occurred together with systemic cytokine alterations and PFC cytokine and microglial changes, with some MIA-associated effects differing in direction or magnitude between female and male offspring. Thus, this study reframes MIA as a model that not only affects brain-related outcomes but also leaves a durable mucosal immune-microbial imprint that may help guide future causal studies of gut-brain immune communication.

## 2 Materials and methods

### 2.1 Animals and experimental design

Wild-type BALB/cByJ mice were obtained from Charles River Laboratories (Barcelona, ES). The animals were maintained in the animal facility of the Faculty of Medicine of the University of Coimbra according to EU and Portuguese legislation (Directive 2010/63/EU; DL113/2013, August 7) under controlled light (12h day/night cycle), temperature and humidity (45%–65%), with *ad libitum* access to hard pellets and water. All the procedures were in accordance with the ethical standards of the Animal Welfare Committee of the Center for Neuroscience and Cell Biology and Faculty of Medicine, University of Coimbra, and the researchers received adequate training and certification from Portuguese authorities (Direção Geral de Veterinária) before the experiments began.

To determine the impact of MIA on neurodevelopment, confirmed pregnant females were injected with 0.05 mg/kg of LPS (lipopolysaccharide derived from *Escherichia coli* O26:B6, Sigma-Aldrich, St Louis, MO, USA; Cat. No. L2654) on gestational day 12.5 to simulate a bacterial infection. This dose was selected to induce a low-grade, transient maternal inflammatory response sufficient to activate the immune system without causing severe maternal illness or fetal loss (21–23). Pregnant females injected with 1× PBS served as controls. At GD13.5, blood from the dams was collected from the tail to ensure an inflammatory profile 24h after injection. Body weight was monitored weekly throughout the study and recorded again immediately prior to euthanasia. Results were expressed as body weight (g). Following the weaning period, the dams were euthanized with an intraperitoneal injection of sodium pentobarbital (200 mg/kg), and blood samples were collected for further analysis.

Male and female offspring were analyzed separately to account for sex as a biological variable. Within each sex, comparisons were performed between control and MIA-exposed animals to assess the effect of MIA. Sex was therefore considered in the experimental interpretation, but it was not included as an independent factor in the statistical model, as the study was designed to evaluate MIA-associated effects within each sex rather than to test the main effect of sex.

### 2.2 Behavioral testing

Eight-week-old males and females were submitted to a series of behavioral tests to evaluate cognitive functions and social dynamics. Prior to testing, the animals were acclimated overnight in the behavior testing room.

Open field test: To assess locomotion activity and hyperactivity-like behavior, animals were placed in an open field arena measuring 42 cm wide × 42 cm deep × 42 cm high. Mice were individually placed at the center of the open field arena for a 30-min trial, which was recorded for analysis. Total distance traveled and velocity were measured using ezTrack, an open-source video analysis tool (24). The floor in the maze was illuminated at 100 lux.

Elevated plus maze test: Anxiety-like behavior was assessed using the Elevated Plus Maze test. The apparatus consisted of two opposing open arms and two opposing closed arms (30 cm long × 5 cm wide), with the closed arms surrounded by 15 cm high walls, all elevated 40 cm above the ground (Open Science, Cat. No. TS0502-M3). Each animal was placed at the center of the arena and allowed to explore freely between the arms for 5 min. Time spent in the open and closed arms, as well as the number of entries into each arm, was quantified using Behavioral Observation Research Interactive Software (BORIS) software (25). The maze floor was illuminated at 20–30 lux.

Tail suspension test: Mice were suspended upside down by the tail using tape of similar length for 6 min, and total immobility time (periods during which the animal remained motionless) was quantified using BORIS software (25). Immobility time exceeding that of control animals was considered indicative of despair-like behavior. The floor of the arena was illuminated at 20–30 lux.

Spontaneous T-maze test: The arena consisted of a T-shaped maze with a start alley (30 cm long × 10 cm wide) and two goal arms (30 cm long × 10 cm wide), each with 20-cm high walls (Open Science, Cat. No. TS0701-M). Animals were placed at the start of the alley and allowed to explore the maze freely. Each trial was recorded to calculate the percentage of alternation. For each animal, five trials (two runs each) were conducted over two consecutive days, with three trials on the first day and two on the second. The maze floor was illuminated at 20–30 lux.

Three-Chamber Social test: Social behavior was assessed using a three-chamber arena (Open Science, Cat. No. TS1701-M). The test consisted of two sessions: in the first session, the animal was placed in the arena for a 20 min habituation period, during which it could freely explore all three chambers. In the second session, the animal was confined to the center chamber while an empty wire cage (empty - “E”) and a cage containing an unfamiliar mouse (stranger 1 - “S1”) were placed in the side chambers. After this, the animal was allowed to explore the chambers containing the “Empty” cage and the “Stranger 1” cage for 20 min. All sessions were recorded, and the time spent in close proximity to the wire cages was quantified using BORIS software (25). Stimulus mice were of the same sex and age as the test animals and had been previously habituated to the wire cages. The maze floor was illuminated at 20–30 lux.

### 2.3 Tissue collection

Animals aged 8–10 weeks were euthanized for tissue collection. Deep anesthesia was induced using an intraperitoneal injection of sodium pentobarbital (200 mg/kg). Blood was collected directly from the heart with an EDTA-K_2_-coated syringe to prevent coagulation. Subsequently, the animals were transcardially perfused with 1× PBS, pH 7.4, followed by 25 ml of a fixative solution containing 4% paraformaldehyde (PFA) and 0.1% glutaraldehyde in 1× PBS, pH 7.4. The intestine was removed, rinsed in 1× PBS, and divided into different segments (duodenum, jejunum, ileum, and colon) and cryopreserved. Approximately 1 cm of ileum and colon was also fixed in 4% PFA for 24h. Following perfusion with 1× PBS and with the fixative solution, brains were extracted and post-fixed in the same fixative solution for an additional 24h at 4°C. For cryosectioning, the fixed tissues were embedded in OCT (Tissue-Tek, Sakura, Finetek, Cat. No. 4583) and frozen in isopentane cooled with dry ice. Samples were stored at −80°C until cryosectioning or homogenization.

### 2.4 Ileum mucosa-associated microbiota profiling

Ileum mucosa-associated material was collected for 16S rRNA gene amplicon sequencing. Ileum segments were opened longitudinally, washed, and mucosa-associated material was gently scraped from the luminal surface and immediately frozen until DNA extraction. Microbial genomic DNA was extracted using the NZY Soil gDNA Isolation Kit (NZYTech, Cat. No. MB21802), according to the manufacturer’s instructions. DNA concentration and purity were assessed using Nanodrop 2000 (Thermo Fisher Scientific, Waltham, MA, USA). The presence of amplifiable DNA was confirmed by PCR using universal 16S rRNA gene primers, as previously described (26). Microbial community profiling was performed by Illumina MiSeq sequencing at the Next Generation Sequencing Unit, Genoinseq, Cantanhede, Portugal. The V4-V5 hypervariable region of the 16S rRNA gene was amplified using the universal forward primer 515F-Y (5′-GTGYCAGCMGCCGCGGTAA-3′) and reverse primer 926R (5′-CCGYCAATTYMTTTRAGTTT-3′) (27) following a standard sequencing protocol. Raw sequencing reads were processed using mothur v.1.44.1 (28). Briefly, paired-end reads were assembled, quality filtered, screened for ambiguous bases and abnormal read lengths, dereplicated, aligned against Silva reference files release 138 (29), screened for chimeric sequences, and taxonomically classified using the SILVA database. Sequences classified as non-bacterial/non-archaeal, chloroplast, mitochondrial, or unclassified at high taxonomic levels were removed before downstream analysis. Taxonomic tables were generated at the genus level for compositional and statistical analyses. Downstream microbiota analyses were performed in R v.4.0.4 using tidyverse v.1.3.2 (30) for data handling and visualization and phyloseq v.1.42.0 (31) for microbiota data integration, filtering, diversity analysis, and graphical representation. Alpha diversity was assessed using the Shannon diversity index. Statistical comparisons between groups were performed using Wilcoxon rank-sum tests, with Benjamini–Hochberg false discovery rate correction applied when multiple comparisons were performed. For downstream genus-level analyses, low-prevalence taxa were filtered by retaining genera detected in at least 33% of samples within at least one experimental group. Relative abundance tables were used for descriptive community profiling and visualization. Bar plots and pie charts were generated from filtered genus-level relative abundance data. Beta diversity was assessed using Bray–Curtis dissimilarity and visualized by non-metric multidimensional scaling. Differences in overall community composition were tested by permutational multivariate analysis of variance using PERMANOVA. Where applicable, homogeneity of multivariate dispersion was assessed to support interpretation of PERMANOVA results.

Differential abundance analysis was performed using complementary approaches. Exploratory genus-level comparisons were conducted using Wilcoxon rank-sum tests on relative abundance data, with Benjamini–Hochberg correction for multiple testing. Count-based differential abundance analysis was performed using DESeq2 (32) on unrarefied count data, applying the package’s internal size-factor normalization. In parallel, beta-binomial regression-based differential abundance testing was performed using corncob (33), allowing assessment of taxon-level differences while accounting for overdispersion. Taxa were considered significantly different between groups when the adjusted *p*-value or false discovery rate was below 0.05, unless otherwise specified.

### 2.5 Tissue homogenates

To analyze innate immunity and synaptic markers, the four intestinal sections, isolated gut epithelial cells, and the PFC were homogenized in a hypotonic lysis buffer (25 mM HEPES, 2 mM MgCl_2_, 1 mM EDTA, and 1 mM EGTA, pH 7.5), supplemented with 1% Triton X-100, 2 mM DTT, 0.1 mM PMSF, protease inhibitors (cOmpleteTM Mini, EDTA-free Protease Inhibitor Cocktail; Roche, Basel, Switzerland, Cat. No. 0469315900), and phosphatase inhibitors (PhosSTOP Phosphatase Inhibitor Cocktail; Roche, Cat. No. 04906837001). Tissue suspensions underwent three freeze-thaw cycles in liquid nitrogen, with intestinal samples additionally sonicated on ice. All samples were then centrifuged at 17,968 × g for 10 min at 4°C. The supernatants were collected and stored at −80°C. Protein content was quantified using the Pierce™ BCA Protein Assay Kit (Thermo Fisher Scientific, Cat. No. 23227) following the manufacturer’s instructions.

### 2.6 Immunoblotting

To assess inflammatory protein and synaptic marker levels via Western blot, 30 µg of protein was resuspended in 6× sample buffer (4× Tris–Cl/SDS, pH 6.8, 30% glycerol, 10% SDS, 0.6 M DTT, 0.012% bromophenol blue) under reducing conditions. After 5 min of denaturation, the samples were loaded onto appropriate % SDS-polyacrylamide gel and transferred to PVDF membranes according to the manufacturer’s protocol (BioRad, Hercules, CA, USA, Cat. No. 1704273). After transfer, the membranes were blocked in Tris-buffered saline (TBS) containing 0.1% Tween 20 and 3% BSA for 1h at room temperature (RT). After blocking, the membranes were incubated overnight at 4°C with gentle agitation with primary antibodies. The following primary antibodies were used: 1:1000 anti-PSD95 (Abcam, Cat. No. ab2723), 1:2000 anti-VGlut1 (NeuroMab, Davis, CA, USA, Cat. No. 75–066 clone N28/9), 1:1000 anti-Gephyrin (Synaptic Systems, Göttingen, Germany, Cat. No. 147111), 1:2500 anti-VGAT (Synaptic Systems, Göttingen, Germany, Cat. No. 131005), and 1:1000 anti-phospho-STAT3 (pSer727) (Cell Signaling, Danvers, MA, USA, Cat. No. SAB4504540). As a loading control, 1:5000 anti-Vinculin (Millipore, Burlington, MA, USA, Cat. No. MAB3574) and 1:5000 anti-GAPDH (Millipore, Burlington, MA, USA, Cat. No. MAB374) were used. Following primary antibody incubation, the membranes were washed three times (each time for 5 min) with TBS containing 0.1% Tween 20. The membranes were then incubated with the appropriate horseradish peroxidase-conjugated secondary antibody for 2h at RT with gentle agitation. Next, the membranes were washed three times and then incubated for 5 min with an alkaline phosphatase-enhanced chemical fluorescence reagent (ECF) (GE Healthcare, Chicago, IL, USA, Cat. No. RPN3685). Fluorescence from the bands of interest was detected using a ChemiDoc MP Imaging System and quantified with ImageLab Software, both from Bio-Rad. The bands of interest were selected, and background subtraction was performed automatically. Quantitative densities were normalized to control samples and further adjusted based on the housekeeping genes mentioned above.

### 2.7 Cytokine assessment by ELISA assay

To assess inflammatory markers, 25 µg of protein from intestinal, epithelial, and PFC tissue or 50 µl of plasma was used to measure the levels of IL-15 (R&D Systems, Minneapolis, MN, USA, Cat. No. DY447-05), IL-23 (R&D Systems, Cat. No. M2300), IL-22 (R&D Systems, Cat. No. M2200), IL-17 (R&D Systems, Cat. No. M1700-1), IL-1β (R&D Systems, Cat. No. MLB00C), IL-6 (R&D Systems, Cat. No. M6000B-1), IL-10 (R&D Systems, Cat. No. M1000B-1), IFN-γ (R&D Systems, Cat. No. MIF00-1), and NF-κB (Abcam, Cambridge, UK, Cat. No. ab176648) using ELISA kits. Absorbance was measured at 450 nm and 540 nm using a SpectraMax Plus 384 multiplate reader.

### 2.8 Immunofluorescence

Ileum and colon: Cryosections were thawed for 1h at RT, hydrated with 1× PBS for 5 min, and then incubated with a solution containing 10% goat serum (TebuBio, Le Perray-en-Yvelines, France, Cat. No. BMS0050) and 0.25% Triton X-100 in 1× PBS for 1h at RT. After incubation, the slides were drained and incubated with the following primary antibodies in 1×PBS containing 1% goat serum and 0.25% Triton-X-100: 1:200 anti-CD11b (BioRad, Cat. No. MCA711GT) or 1:150 anti-CD8α-PE (Miltenyi Biotec, Bergisch Gladbach, Germany, Cat. No. 130-123-781). The incubation was carried out for 24h at 4 °C. After three 5-min washes with 1× PBS, the slides were incubated for 2h at RT with the following secondary antibodies: 1:300 goat anti-rabbit Alexa Fluor 488 (Life Technologies, Carlsbad, CA, USA, Cat. No. A11008), 1:300 goat anti-rabbit Alexa Fluor 594 (Life Technologies, Carlsbad, CA, USA, Cat. No. A11012), or 1:300 goat anti-rat Alexa Fluor 594 (Life Technologies, Cat. No. A11007). The slides were washed and incubated for 10 min with 1:2000 Hoechst 33342 (Sigma-Aldrich, Cat. No. 14533). Finally, the sections were mounted with Mowiol^©^ (Sigma-Aldrich, St. Louis, MO, USA Cat. No. 81382). A total of eight images were acquired per animal from different ileum/colon sections. Images were captured using a confocal microscope (LSM710, Zeiss) with a Plan-Apochromat 20×/0.8 M27 objective at 1024 × 1024 resolution. For CD8α-specific images, a Plan-Apochromat 40×/1.4 oil DIC M27 objective was used, also at 1024 × 1024 resolution. Intensity analysis was performed using QuPath 0.5.1 software (34).

Prefrontal cortex: The slides were thawed for 1h and hydrated with 1× PBS. For Iba1 staining, antigen retrieval was performed. The slices were incubated with 0.01 M citrate buffer, pH 6.0, and subjected to two cycles of heating for 4 min each. The sections were then quenched with methanol containing 1% hydrogen peroxide for 20 min, followed by incubation with 1× PBS supplemented with 10% goat serum (Sigma-Aldrich, Cat. No. D9663) for 1h at RT. Next, the slices were incubated for 24h at 4°C with a 1× PBS solution containing a 1:500 anti-Iba1 (Wako, Osaka, Japan, Cat. No. 019-19741), 1:500 anti-CD68 (BioRad, Cat. No. MCA1957), or 1:500 anti-C3 (Abcam, Cat. No. ab11862) antibody, 1% goat serum, and 0.25% Triton-X-100. After incubation, the sections were washed with 1× PBS and incubated for 2h at RT with 1:500 goat anti-rabbit AlexaFluor 488 (Life Technologies, Cat. No. A-11006) and goat anti-rat AlexaFluor 595 (Life Technologies, Cat. No. A-11007). The slides were then washed and incubated for 10 min with 1:2000 Hoechst 33342 (Sigma-Aldrich, Cat. No. 14533). Finally, the sections were mounted with Mowiol^©^ (Sigma-Aldrich, Cat. No. 81382). A total of six images per animal from different PFC regions were acquired using a confocal microscope (LSM710, Zeiss) with a Plan-Apochromat 40×/1.4 Oil DIC M27 objective at 1024 × 1024 resolution. IMARIS software (Oxford Instruments) was used to quantify Iba1 signal intensity per volume, assess microglial complexity using Sholl analysis, and determine the colocalization level of CD68 or complement component C3 within Iba1-positive microglial cells.

### 2.9 Intraepithelial lymphocytes isolation

The protocol used was adapted from (35) with slight modifications.

After euthanasia, the small and large intestines of the animals were collected, and all fat and mesentery were removed. During dissection, the tissue was maintained on ice in RPMI-1640 medium (Merck, Cat. No. R1383) and rinsed with 1x PBS. Peyer’s patches were removed and counted. Both segments of the intestine were cut into small pieces using a scalpel and placed into a solution of 1% HBSS (Gibco, Grand Island, NY, USA, Cat. No. 14065-056) supplemented with 1 mM DTT (Nzytech, Cat. No. MB03101), 1 mM EDTA (Sigma-Aldrich^®^), and 5% FBS (Corning, Corning, NY, USA, Cat. No. 35-015-CV). Each sample was vortexed at maximum speed for 30 s, followed by a 20-min incubation at 37°C in a water bath with manual homogenization every 5 min. The cell suspension was then passed through a 100-μm cell strainer, and the homogenization protocol was repeated for the non-homogenized tissue. Finally, the tubes were centrifuged at 300 × g for 5 min at 4°C, and the supernatant was discarded. For optimal separation of IELs from epithelial cells, the pellet was resuspended in 40% Percoll and layered onto an 80% Percoll solution to create a gradient. The samples were then centrifuged at 1,200 × g for 30 min at RT, without brake. After centrifugation, the IELs were found at the interface between the two Percoll concentrations, with the epithelial cells on top. The epithelial cells were stored at −80°C until further use. The IELs were washed with warm media, centrifuged at 700 × g for 5 min, and resuspended in FACS buffer (1× PBS supplemented with 5% FBS, 1M HEPES (Corning, Cat. No. 25-060-Cl), and 10 mg/ml DNAse I (Roche, Cat. No. 10104159001)). The IELs were then ready for staining and subsequent flow cytometry.

### 2.10 Flow cytometry of IELs from intestinal tissue and whole blood

The IEL suspensions were incubated with a 1:400 anti-TCRβ-FITC (BioLegend, San Diego, CA, USA, Cat. No. 109206), 1:200 anti-TCRδ-PE (BioLegend, Cat. No. 118108), 1:200 anti-CD8β-PerCP/Cy5 (BioLegend, Cat. No. 126610), 1:200 anti-CD8α-APC/Cy7 (BioLegend, Cat. No. 100713), 1:400 anti-CD4-APC (Miltenyi Biotec, Cat. No. 130-116-487), and 1:50 Live/Death Aqua (Thermo Fisher Scientific, Cat. No. L34957) for 15 min on ice and protected from light. After incubation, stained cells were washed with FACS buffer and centrifuged at 300 × g for 2 min at 10°C. The supernatant was discarded, FACS buffer was added, and it was transferred to suitable flow cytometry tubes. The IEL samples were then ready for analysis using a BD FACSCanto II flow cytometer.

To determine the subpopulation of IELs in whole blood, a 50-μl peripheral blood sample was incubated with 1 μl of each antibody mentioned above. After 15 min incubation at RT, 2 ml of lysis buffer (Invitrogen, Cat. No. TM 00-4333-57) was added, and samples were incubated again for an additional 15 min. Samples were centrifuged at 400 × g for 5 min twice, and the supernatant was discarded. Finally, 200 μl of 1× PBS was added to the pellet. To determine CD4 and CD8 populations in whole blood, 50 μl of blood samples were incubated with 1 μl of anti-CD45-PerCP (Miltenyi Biotec, Cat. No. 130-102-469), anti-CD3-FITC (Miltenyi Biotec, Cat. No. 130-119-798), anti-CD4-APC (Miltenyi Biotec, Cat.No. 130-116-487) and anti-CD8a-PE (Miltenyi Biotec, Cat. No. 130-122-017) with the same protocol as described above. Samples were ready to be analyzed with a BD FACSCanto II flow cytometer (BD Biosciences, San Jose, CA, USA). Flow cytometry data was analysed using FlowJo V10 (BD Biosciences).

### 2.11 Data analysis

Data are presented as means ± standard error of the mean (SEM). Statistical analyses, using GraphPad Prism 8.4.0 for Windows (GraphPad Software), were performed according to the biological comparison addressed by each experiment. Sample normality was assessed using the Shapiro–Wilk’s test to determine the appropriate parametric or non-parametric tests. Since males and females were analyzed as distinct biological subgroups, the primary comparison tested was the effect of MIA within each sex. Accordingly, the Unt and MIA groups were compared separately in females and males using an unpaired Student’s t-test or Mann–Whitney U test, depending on data distribution. When comparison of all four experimental groups was required, data were analyzed by ordinary one-way analysis of variance (ANOVA) followed by Tukey’s multiple comparisons test or by Kruskal–Wallis test followed by Dunn’s test for non-parametric data. These analyses were used to compare specific experimental groups and were not interpreted as formal tests of sex × MIA interaction. Microbiome data analysis is described above. A *p*-value < 0.05 was considered statistically significant. A summary table (**Supplementary Table 1**) listing exact n per group per assay is available in the **Supplementary Materials**.

## 3 Results

### 3.1 Experimental design and maternal response to low-dose LPS-MIA

Pregnant BALB/cByJ females received *E. coli*–derived LPS (0.05 mg/kg) at GD12.5 or PBS, and male and female offspring were analyzed in adulthood (10 weeks) using a multi-compartment design (**Supplementary Figure 1A**). Offspring body weight did not differ between PBS and MIA groups within sex at the analyzed age (**Supplementary Figure 1B**). Maternal blood was collected at GD13.5 and after weaning. LPS injection, at GD13.5, induced an increase in plasmatic IL-6 levels across dams but no alteration in IL-17 levels (**Figure 1A**). Post-weaning dams’ plasma IL-17 values increased (**Figure 1A**).

**Figure 1:**
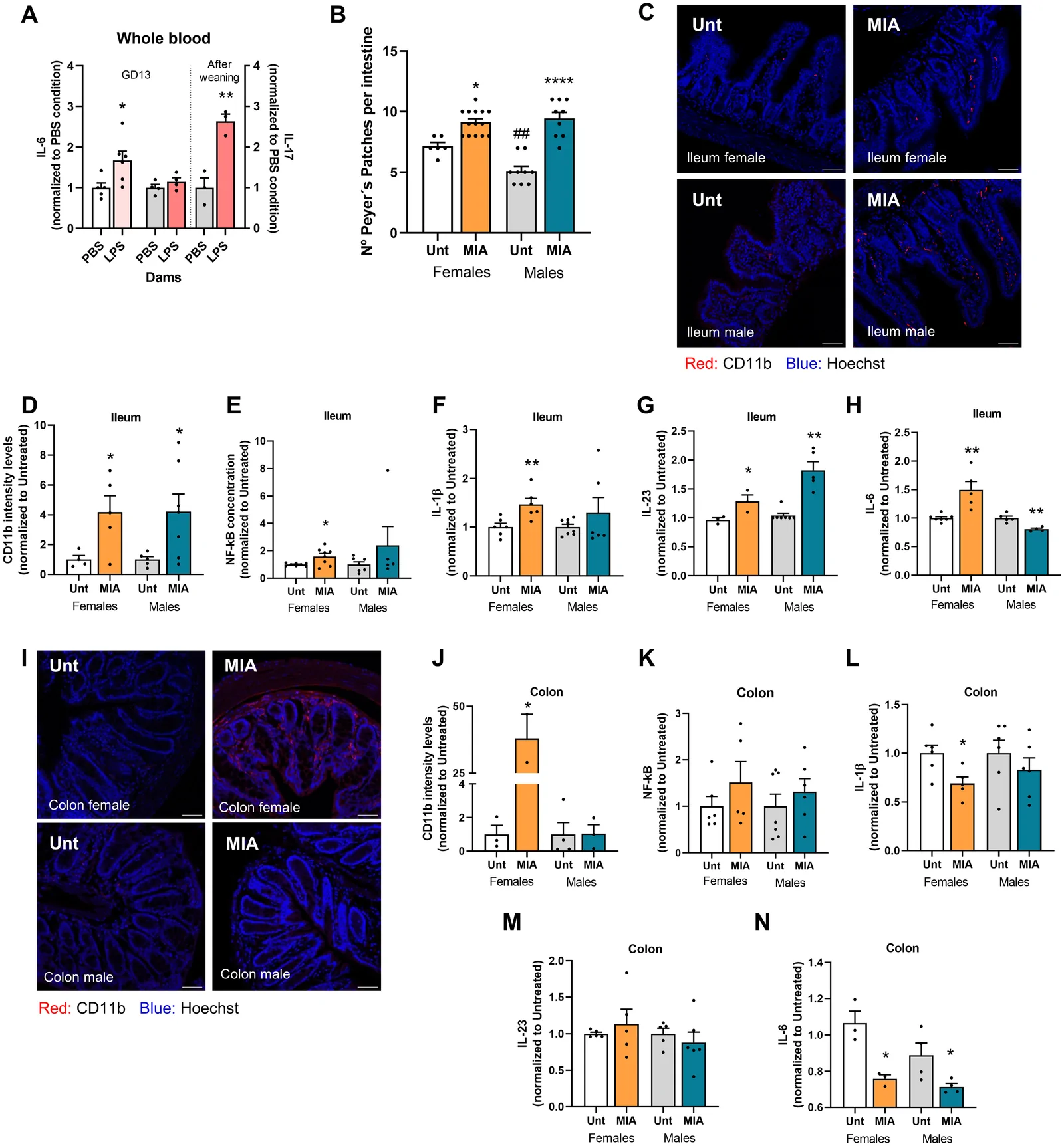
MIA is associated with region-specific ileal immune activation. **(A)** IL-6 and IL-17 evaluation by ELISA assay in whole blood of dams collected at gestational day 13.5 and after weaning (*n* = 3–6 mice per group). **(B)** Peyer’s Patches count per intestine in MIA animals (*n* = 7–17 mice per group). **(C)** Representative immunofluorescence images of mouse ileum sections stained with anti-CD11b and **(D)** respective quantification (*n* = 4–6 mice per group). Assessment of **(E)** NF-κB, **(F)** IL-1β, **(G)** IL-23, and **(H)** IL-6 levels in ileum of MIA animals by ELISA (*n* = 3–8 mice per group). **(I)** Representative immunofluorescence images of mouse colon sections stained with anti-CD11b and **(J)** respective quantification (*n* = 3–4 mice per group). Assessment of **(K)** NF-κB, **(L)** IL-1β, **(M)** IL-23, and **(N)** IL-6 levels in colon of MIA animals by ELISA (*n* = 3–7 mice per group). *p < 0.05; **p < 0.01; ****p < 0.0001; statistical comparisons were conducted between Unt and MIA conditions, separately for females and males using Unpaired t-test or **(B)** between all conditions using one-way ANOVA followed by Tukey’s multiple comparisons test. ^##^*p* < 0.01; statistical differences between Unt conditions. Results are presented as the mean ± SEM. Scale bars are 25 µm. See also **Supplementary Figure 1**.

### 3.2 MIA is associated with a region-specific ileal inflammatory profile

The Peyer’s patch number in the small intestine increased in MIA (**Figure 1B**), suggesting an alteration in gut immune development and intestinal immunity regulation. Analysis of intestinal epithelial cell (IEC) fractions showed reduced IL-15 in the small intestine of MIA females and reduced IL-23 in the small intestine of both sexes (**Supplementary Figures 1C**, **E**), whereas colonic epithelial IL-15 levels did not change (**Supplementary Figure 1D**). IL-23 was reduced in the colon of MIA male offspring, while it showed a non-significant increase in females (*p* = 0.0657) (**Supplementary Figure 1F**). These findings indicate altered epithelial cytokine profiles but do not establish that epithelial cells are the source of downstream immune-cell recruitment.

CD11b intensity was increased (**Figures 1C, D**), which is consistent with ileal activation of NF-κB in both MIA male and female offspring (**Figure 1E**), indicating an upregulation of inflammatory pathways. Indeed, IL-1β (**Figure 1F**) and IL-23 (**Figure 1G**) levels increased in both MIA male and female offspring. Ileal IL-6 showed divergent patterns within each sex, increasing in females and decreasing in males (**Figure 1H**). In contrast, the colon showed a less consistent inflammatory profile. CD11b intensity increased mainly in females (**Figures 1I, J**), NF-κB showed only a trend (**Figure 1K**), IL-23 was unchanged (**Figure 1M**), and IL-1β (**Figure 1L**) and IL-6 were reduced in both sexes (**Figure 1N**). These data support regional immune heterogeneity rather than uniform gut inflammation.

### 3.3 MIA remodels gut IEL composition in female and male offspring

In our study, we characterized IEL populations in the small intestine of MIA mice (**Supplementary Figures 2A**–**C**), since their differentiation and function may likely be influenced by the elevated IL-23 levels (**Figure 1G**). IEL profiling revealed changes in both TCRγδ and TCRαβ compartments (**Figures 2A, B**; gating in **Supplementary Figure 2B**). MIA was associated with reduced TCRγδ double-negative IELs and increased TCRγδ CD8αα IELs in both sexes (**Figure 2A**). TCRαβ IELs showed distinct patterns in female and male offspring. Untreated males exhibited lower frequencies of TCRαβ CD8αβ IELs than untreated females, while MIA increased this population in males, with no significant MIA-associated change detected in females (**Figure 2B**). Both sexes showed reduced TCRαβ double-negative IELs (**Figure 2B**). These findings identify IEL remodeling as a central and previously underexplored feature of the MIA-induced intestinal phenotype.

**Figure 2:**
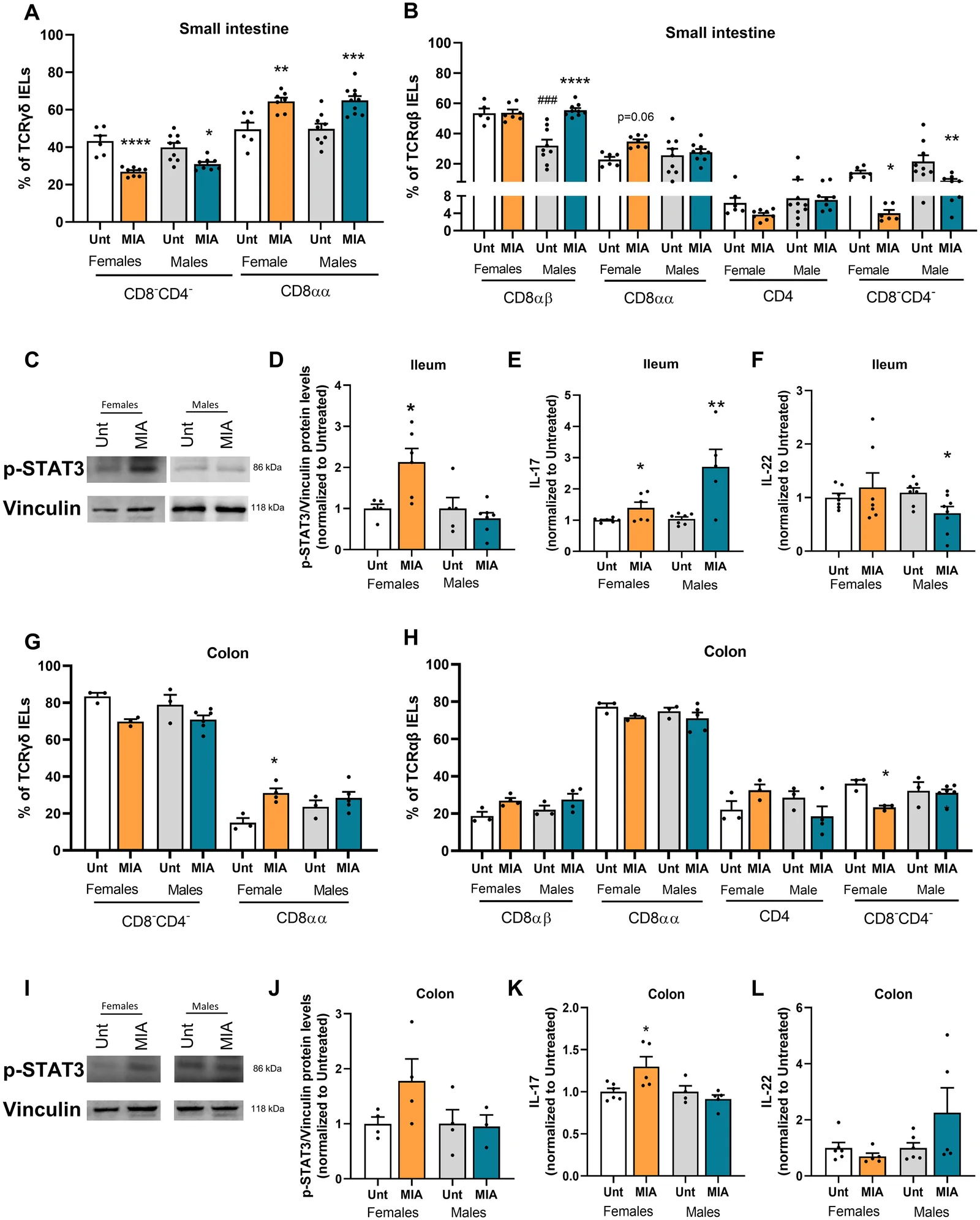
MIA remodels small-intestinal IEL subsets and IL-17/IL-22–associated mucosal cytokines. **(A, B)** Isolation and analysis of Intraepithelial Lymphocytes cells (IELs) subsets from the small intestine by flow cytometry (*n* = 6–10 mice per group). **(C)** Representative immunoblot for p-STAT3 levels in ileum samples. The blots were reprobed for vinculin to confirm equal protein loading. **(D)** Densitometric analysis of p-STAT3 levels normalized to vinculin measured by Western Blot (*n* = 5–7 mice per group). Assessment of **(E)** IL-17, **(F)** IL-22 levels ileum of MIA offspring by ELISA (*n* = 4–8 mice per group). **(G, H)** Isolation and analysis of IELs subsets from colon by flow cytometry (*n* = 3–5 mice per group). **(I)** Representative immunoblot for p-STAT3 levels in colonic samples. The blots were reprobed for vinculin to confirm equal protein loading. **(J)** Densitometric analysis of p-STAT3 levels normalized to vinculin measured by Western Blot (*n* = 3–4 mice per group). Assessment of **(K)** IL-17, **(L)** IL-22 levels colonic of MIA offspring by ELISA (*n* = 3–6 mice per group). *p < 0.05; **p < 0.01; ***p < 0.001; ****p < 0.0001; statistical comparisons were conducted between Unt and MIA conditions, separately for females and males using Unpaired t-test or **(A, B, G, H)** between all conditions using one-way ANOVA followed by Tukey’s multiple comparisons test. ^###^*p* < 0.001; statistical differences between Unt conditions. Results are presented as the mean ± SEM. See also **Supplementary Figure 2**.

MIA female offspring showed an increase in phosphorylated STAT3 (pSTAT3) (**Figures 2C, D**), indicating enhanced STAT3-driven pathways in these immune cells. We observed an increase in IL-17 levels in the ileum of MIA progeny (**Figure 2E**), whereas ileal IL-22 was reduced in MIA males (**Figure 2F**), suggesting an upregulation of pro-inflammatory responses. IL-10 and IFN-γ were not changed in the ileum (**Supplementary Figures 2D**, **E**). Together, these data reveal an IL-17/IL-22-associated mucosal immune signature that differs by sex, while not establishing IELs as the cellular source of these cytokines.

Colonic IEL analysis showed an increase in TCRγδ CD8αα cells in females (**Figure 2G**). Female MIA offspring also showed a reduction in TCRαβ double-negative cells in the colon, whereas males did not show colonic IEL changes (**Figure 2H**). Colonic pSTAT3 was not altered (**Figures 2I, J**). In the colon IL-17 increased in females, and IL-22 increased in males (**Figures 2K, L**). Interestingly, IL-10 increased in MIA male offspring (**Supplementary Figure 2I**), whereas IFNγ increased in MIA female offspring (**Supplementary Figure 2J**). These results are consistent with mucosal immune remodeling that varies by region and sex, with stronger and more integrated changes observed in the ileum.

### 3.4 MIA reshapes the ileal mucosa-associated microbiota, with enrichment of SFB and depletion of *Lactobacillus*

We next performed 16S rRNA gene sequencing of ileal mucosa-associated bacterial communities to determine whether MIA was associated with long-term microbial alterations at the epithelial interface. Across all samples, we detected 307 OTUs, assigned to 134 genera, 89 families, 60 orders, 27 classes, and 17 bacterial phyla. Shannon alpha diversity was not significantly altered, although a trend toward increased diversity was observed in both male and female MIA offspring (**Figure 3A**). Bray-Curtis ordination suggested MIA-associated changes in ileal community structure, with a more pronounced separation in females (**Figure 3B**). Taxonomic profiling and differential abundance analyses identified selected MIA-associated changes in the ileal mucosa-associated microbiota (**Figures 3C, D**). Interestingly, MIA offspring exhibited increased relative abundance of segmented filamentous bacteria (SFB) and *Bacteroides* in both sexes (**Figures 3E, F**). On the other hand, *Lactobacillus* relative abundance was reduced in the ileal mucosa of MIA offspring (**Figure 3G**). Given the established immunomodulatory roles of SFB as potent inducers of intestinal Th17 cells (13) and of *Lactobacillus* as promoters of regulatory/protective mucosal responses (36), these findings suggest that MIA is associated with persistent remodeling of ileal microbial communities, with likely consequences for mucosal immune tone.

**Figure 3:**
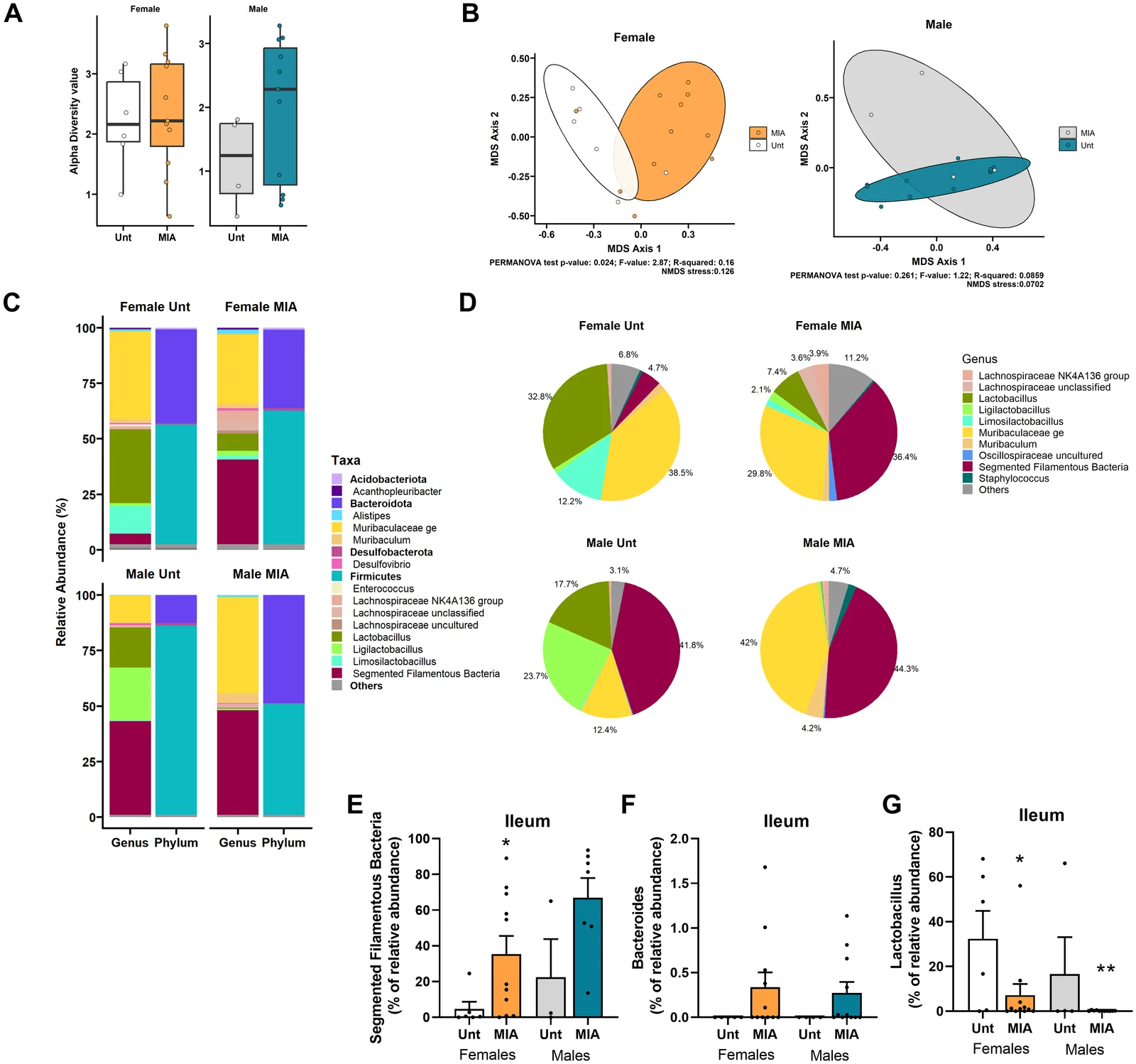
MIA alter mucosa-associated ileal microbiota. **(A)** Alpha diversity measured using the Shannon index at the OTU level, derived from 16S rDNA sequences obtained from mucosa-associated ileal samples (*n* = 4–11 mice per group). **(B)** Beta diversity evaluated by Principal Coordinate Analysis (PCoA) based on the Bray-Curtis dissimilarity index of OTUs derived from 16S rDNA sequencing of mucosa-associated ileal samples (*n* = 4–11 mice per group) analyzed by PERMANOVA with DESeq2 Corncob Wald test statistical analysis. **(C, D)** Taxonomic composition of ileal mucosa-associated microbiota at the phylum and genus levels (*n* = 4–11 mice per group). Relative abundance of **(E)** Segmented Filamentous Bacteria, **(F)** *Bacteroides* and **(G)** *Lactobacillus* associated with the ileal mucosa of the animals (*n* = 3–11 mice per group). *p < 0.05; statistical comparisons were conducted between Unt and MIA groups, separately for females and males using the Wilcoxon test with Benjamini–Hochberg correction for multiple comparisons. Results are presented as the mean ± SEM.

### 3.5 Systemic immune changes show shared IL-6 and female-biased IL-17 responses

Whole-blood immune profiling revealed systemic changes that were not identical between sexes. Circulating lymphocyte frequency increased in MIA females (**Supplementary Figure 3A**, **B**), whereas CD4 and CD8 populations decreased in females (**Figures 4A, B**). Although the percentage of TCR cells remained constant in MIA offspring (**Supplementary Figure 3C**), selected TCRγδ CD8αα and TCRαβ double-negative subsets were reduced in the blood of both (**Figures 4C, D**). Plasma cytokine analysis showed increased IL-6 in both MIA offspring (**Figure 4E**), whereas IL-17 increased in females and decreased in males (**Figure 4F**). These results indicate a shared systemic IL-6 response with a sex-divergent IL-17-associated profile, with a specific increase observed only in females.

**Figure 4:**
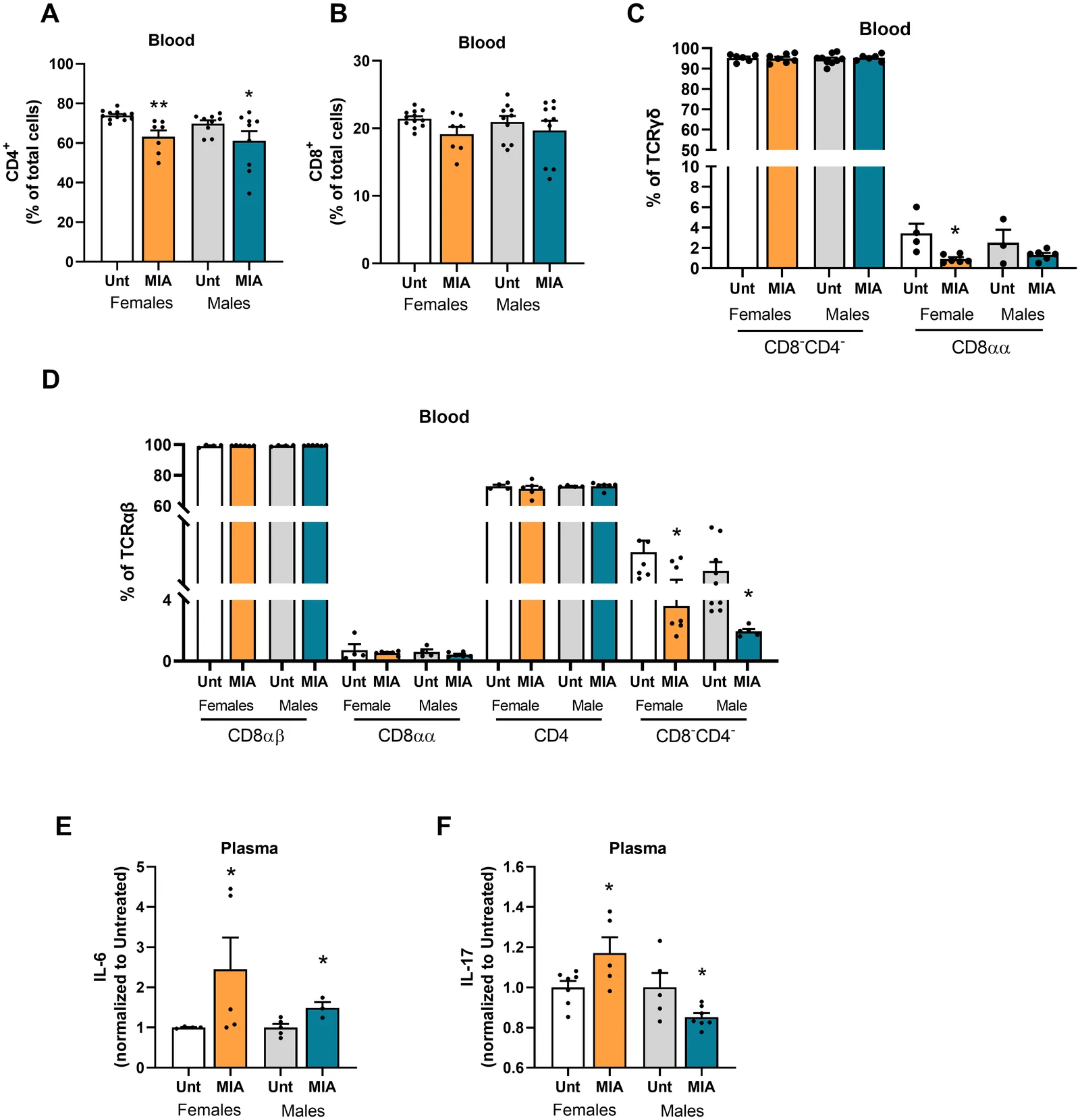
Systemic immune changes reveal shared IL-6 and female-biased IL-17 responses. **(A)** CD4, **(B)** CD8 cell percentage in whole blood obtained by flow cytometry (*n* = 7–12 mice per group). **(C, D)** Analysis of TCR lymphocyte subsets from whole blood by flow cytometry (*n* = 3–9 mice per group). Assessment of **(E)** IL-6 and **(F)** IL-17 levels in plasma samples of MIA offspring by ELISA (*n* = 4–7 mice per group). *p < 0.05; **p < 0.01; ***p < 0.001; statistical comparisons were conducted between all conditions using **(A)** Kruskal–Wallis test followed by Dunn’s multiple condition test or **(C, D)** using one-way ANOVA followed by Tukey’s multiple comparisons test. **(E, F)** Statistical comparisons were conducted between Unt and MIA conditions, separately for females and males using Mann Whitney test or Unpaired t-test. Results are presented as the mean ± SEM. See also **Supplementary Figure 3**.

### 3.6 PFC microglial profiles and cytokine levels diverge by sex after MIA

We then assessed whether gut and systemic immune alterations co-occurred with PFC cytokine and microglial morphological and activation-associated changes. Iba1 intensity per volume increased in female MIA offspring compared with female controls, whereas male MIA offspring displayed lower values than female MIA offspring but did not differ from male controls (**Figures 5A, B**). Microglial filament length and Sholl analysis revealed baseline differences and divergent MIA-associated morphological responses between males and females. Control males displayed higher Sholl intersections than control females at selected radial distances. Following MIA, females showed increased filament length and a broader Sholl intersection profile, consistent with enhanced arborization, whereas males showed reduced filament length and a downward shift in Sholl intersections relative to male controls, suggesting simplified microglial morphology (**Figures 5C–E**; **Supplementary Figure 4A**). Microglial number and density increased in MIA female offspring (**Supplementary Figures 4**, **S5B, C**), suggesting that the MIA-associated microglial phenotype may involve changes in cell abundance as well as morphology. Additionally, we characterized microglial activation states by colocalization of CD68 or C3 in Iba1^+^ cells (**Figures 5F–H**). We observed increased colocalization of C3 (**Figures 5F, G**) or CD68 (**Figures 5H, I**) within Iba1^+^ cells in males, indicating that in these animals’ microglia may be activated and exhibit a more phagocytic/reactive phenotype. In females, microglia showed elevated C3, without changes in CD68, suggesting a more subtly altered phenotype. Interestingly, C3 levels within Iba1^+^ cells correlated positively with ileal mucosa-associated SFB in both sexes (**Figure 5J**).

**Figure 5:**
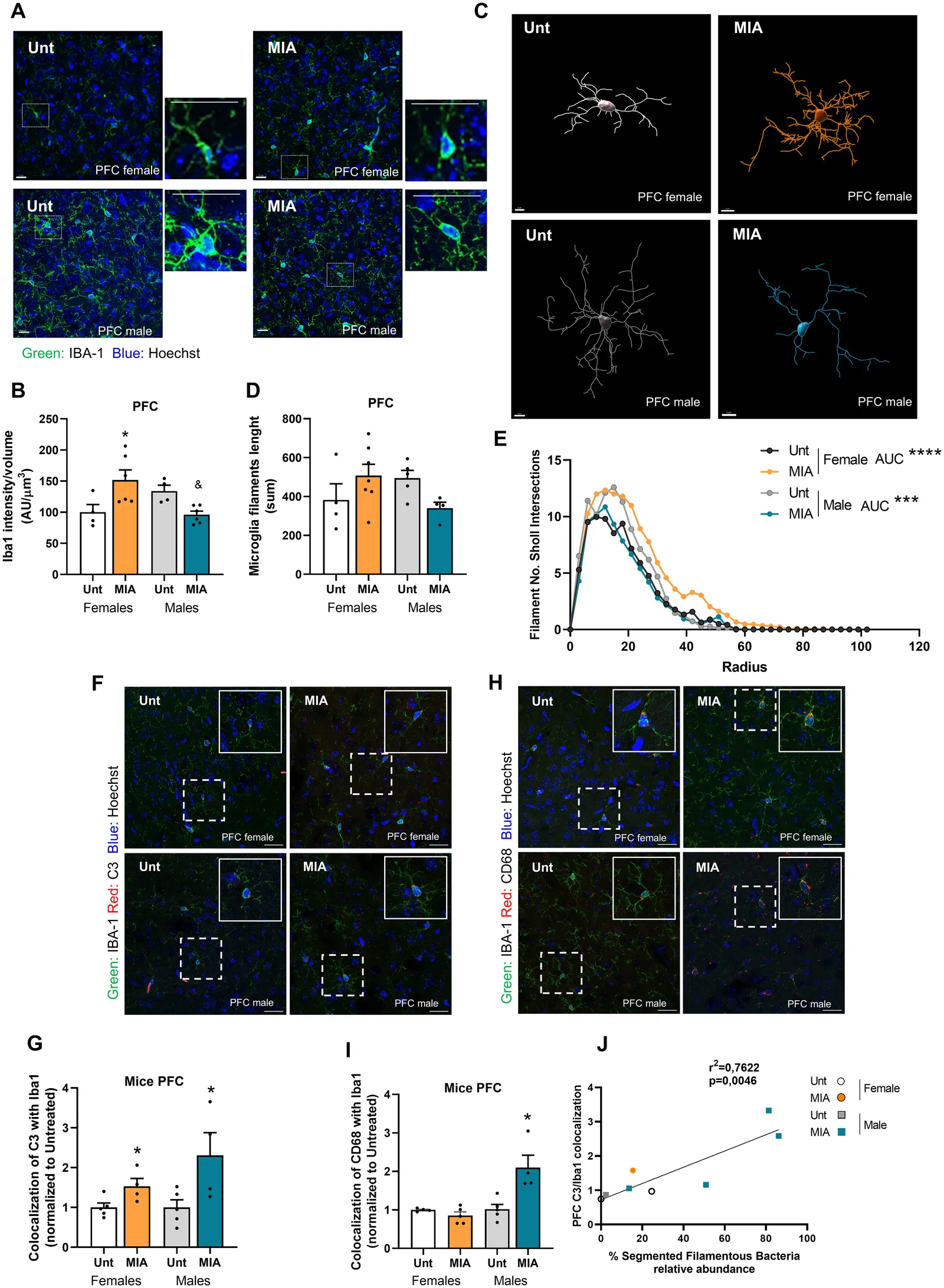
Effects of MIA on PFC cytokine profiles and microglial morphology in female and male offspring. **(A)** Representative immunofluorescence images of PFC sections stained with anti-Iba1 and **(B)** respective quantification (*n* = 4–7 mice per group). Scale bars are 15 µm. **(C)** Representative image of microglia reconstitution, **(D)** respective microglia filaments length quantification and **(E)** Sholl analysis using IMARIS software (*n* = 4–7 mice per group). Scale bars are 5 µm. **(F, G)** Colocalization of C3 with Iba1-positive microglial cells. **(H, I)** Colocalization of C3 with Iba1-positive microglial cells. Scale bars are 25 µm. **(J)** Pearson Correlation between ileal mucosa-associated SFB and C3/Iba1 colocalization in PFC (*n* = 8 mice, both sexes are pooled). *p < 0.05; ***p < 0.001; ****p < 0.001; statistical comparisons were conducted between all conditions **(B)** using one-way ANOVA followed by Tukey’s multiple comparisons test. **(E, G, I)** Statistical comparisons were conducted between Unt and MIA conditions, separately for females and males using Unpaired t-test. & *p* < 0.05; statistical differences between MIA conditions. Results are presented as the mean ± SEM. See also **Supplementary Figure 4**.

We also examined STAT3 activation and the associated cytokine profile in the PFC of MIA offspring, revealing further differences between sexes. PFC pSTAT3 levels increased in females, indicating activation of the STAT3 signaling pathway with a marked increase in IL-17 levels (**Figures 6A–C**). In contrast, MIA male progeny did not show an increase in pSTAT3 levels in the PFC, suggesting a lack of strong STAT3-driven inflammatory responses in the male brain (**Figures 6A, B**). Instead, we observed an increase in IL-22 levels in male offspring (**Figure 6D**). NF-κB and IL-1β were not increased in the PFC (**Supplementary Figures 4D, E**). These data demonstrate MIA-associated changes in PFC cytokine profiles and microglial reactivity in female and male offspring, including morphological remodeling and altered Iba1-associated C3 and CD68 immunoreactivity. However, the current experiments do not identify the cellular source of PFC IL-17 or IL-22.

**Figure 6:**
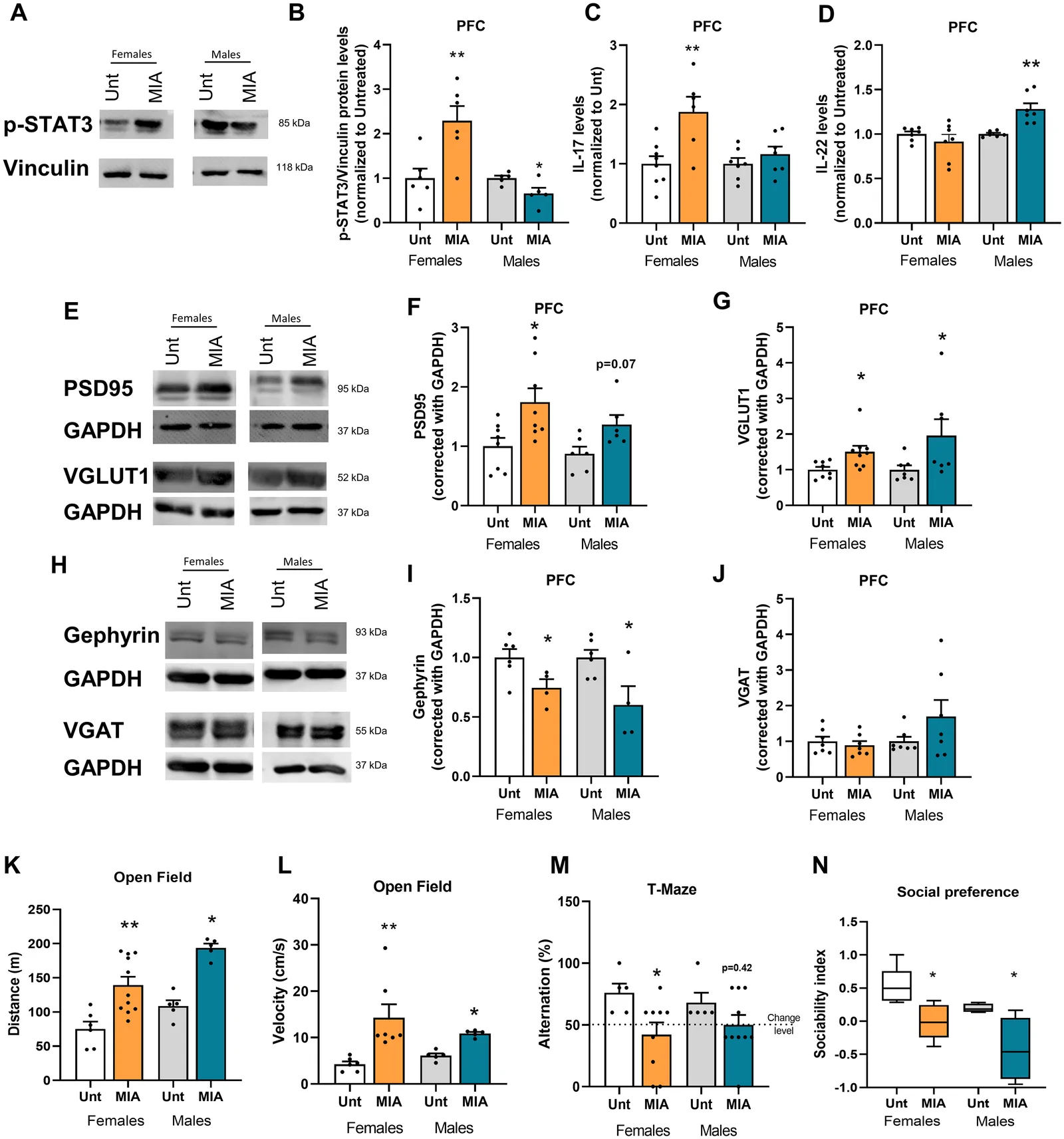
Behavioral and synaptic marker alterations after MIA. **(A)** Representative immunoblot for p-STAT3 levels in PFC samples. The blots were reprobed for vinculin to confirm equal protein loading. **(B)** Densitometric analysis of p-STAT3 levels normalized to vinculin measured by Western Blot (*n* = 5–6 mice per group). Assessment of **(C)** IL-17 and **(D)** IL-22 levels in the PFC of MIA offspring by ELISA Blot (*n* = 6–8 mice per group). **(E)** Representative immunoblot and densitometric analysis of PFC samples and respective quantification of **(F)** PSD95, **(G)** VGLUT1 protein levels. **(H)** Representative immunoblot and densitometric analysis of PFC samples and respective quantification of **(I)** Gephryin, **(J)** VGAT protein levels (*n* = 4–9 mice per group). **(K, L)** Hyperactive behavior measured by distance traveled and velocity parameters in Open Field test (*n* = 5–11 mice per group). **(M)** T-maze test to evaluate working memory (*n* = 5–11 mice per group). **(N)** Sociability index in Three-Chamber Social test to assess sociability (*n* = 4–6 mice per group). *p < 0.05; **p < 0.01; statistical comparisons were conducted between Unt and MIA conditions, separately for females and males using Unpaired t-test. Results are presented as mean ± SEM. See **Supplementary Figure 3** also.

### 3.7 Behavioral and prefrontal cortex synaptic readouts indicate neurodevelopmental vulnerability

To understand how microglial alteration correlated with MIA progeny behavior, we determined the excitatory and inhibitory postsynaptic balance in the PFC. PFC synaptic protein analysis showed increased PSD95 (postsynaptic density protein 95) and VGLUT1 (vesicular glutamate transporter 1) levels and reduced gephyrin levels in MIA offspring, with VGAT (vesicular GABA transporter) showing no alteration (**Figures 6E–J**). These protein-level changes are consistent with altered excitatory/inhibitory synaptic marker balance (**Supplementary Figures 5A**, **B**) but do not directly measure synaptic transmission. Behavioral testing identified increased locomotor/exploratory activity in the open field in both sexes (**Figures 6K, L**; **Supplementary Figure 5C**). In the spontaneous T-maze, MIA offspring showed reduced alternation performance, consistent with altered working memory or cognitive flexibility (**Figure 6M**). MIA offspring showed reduced social preference in the three-chamber test, indicating altered sociability or reduced social approach behavior (**Figure 6N**). Tail suspension (**Supplementary Figure 5E**) and elevated plus maze outcomes (**Supplementary Figures 5F**–**I**) should be interpreted in relation to locomotor activity, because reduced immobility or increased open-arm time may reflect hyperactivity/exploration rather than reduced despair or anxiety. Overall, these behavioral outcomes indicate altered neurodevelopmental-related behavioral dimensions, not a definitive SCZ-like syndrome.

## 4 Discussion

This study identifies the ileal mucosal interface as a persistent site of alteration in adult offspring exposed to low-dose LPS-induced MIA. MIA models are useful for studying how prenatal inflammation modifies neurodevelopmental risk, but their outcomes depend on the inflammatory stimulus, dose, gestational timing, strain, and sex-related biological context (3, 19, 37, 38). Within this context, our data show that MIA was associated with stronger immune changes in the ileum than in the colon, suggesting a regionally restricted mucosal imprint. This extends previous work showing that MIA can alter offspring intestinal physiology and fecal gut composition (16) and supports the idea that prenatal inflammation may influence later intestinal immune susceptibility (17).

Our data identify IEL remodeling as a prominent feature of the intestinal immune alterations observed in adult MIA offspring. IELs are strategically positioned at the epithelial interface, where they contribute to mucosal surveillance, tissue adaptation, and immune regulation (10, 11, 39). MIA was associated with changes in TCRγδ and TCRαβ IEL subsets, including TCRγδ CD8αα or CD8^-^CD4^-^ populations and TCRαβ CD8αβ or CD8^-^CD4^-^ cells. These alterations are relevant since γδ IELs respond rapidly to epithelial stress and support mucosal homeostasis (40), while TCRαβ DN and CD4 IEL subsets may contribute to immune regulation and tolerance-related responses (41–43). The increase in TCRαβ CD8αβ cells observed in males may also indicate a shift toward a more cytotoxic IEL profile, although this requires functional validation (44).

These IEL changes occurred together with altered ileal cytokine signaling, including IL-17/IL-22–associated readouts. IL-23 can promote IL-17–related mucosal responses and influence IEL activity under epithelial stress or inflammatory conditions (11, 45, 46). STAT3-associated signaling is also relevant in this context, as IELs and other mucosal immune cells can produce cytokines such as IL-17, IL-22, IL-10, and IFN-γ depending on activation state and tissue context (47–49). However, the present flow cytometry panel defines IEL phenotype rather than function, and the cellular source of IL-17 or IL-22 was not directly established.

The mucosa-associated microbiota data provides a microbial context for the IEL phenotype. MIA was associated with altered ileal mucosa-associated microbiota composition, including shifts in segmented filamentous bacteria (SFB), *Bacteroides* and *Lactobacillus*. This is particularly relevant because mucosa-associated bacteria reside in close proximity to epithelial and immune cells, placing them in a privileged position to influence host-microbe immune signaling. Among these taxa, SFBs are of special interest because they adhere closely to intestinal epithelial cells and promote Th17-related mucosal programmes (13, 50–52), and have also been linked to IEL modulation (14, 15). Importantly, SFB has also been identified as a key determinant of MIA susceptibility. In pregnant dams, SFB-driven expansion of intestinal Th17 cells calibrates the magnitude of the maternal IL-17A response to inflammatory triggers, which in turn leads to neurodevelopmental and behavioral abnormalities in offspring (53). This is consistent with our findings since SFB abundance was increased in the ileal mucosa of both male and female MIA offspring, paralleling the increase in ileal IL-17 observed in both sexes, supporting a potential link between SFB colonization and local Th17-associated mucosal immune responses due to MIA. *Bacteroides* can influence regulatory and Th17-related immune responses depending on species and context (54), whereas *Lactobacillus* taxa are often associated with anti-inflammatory and barrier-supportive effects, although these actions are strain-dependent (55). Since offspring microbiota composition is strongly influenced by maternal microbial transmission during and after birth (56), we cannot exclude the possibility that postnatal colonization from the dam contributes to the microbiota alterations observed in MIA offspring. The systemic and intestinal immune alterations observed in MIA offspring raise the possibility that prenatal immune activation may alter the developing gut environment and thereby influence subsequent microbial colonization and community structure. Together, these findings support a model in which MIA is associated with coordinated changes at the mucosa-associated microbiota–IEL interface, while the present study cannot distinguish the relative contributions of maternal microbial transmission and offspring immune remodeling or establish the temporal direction of these changes.

The systemic and PFC analyses further indicate that this mucosal phenotype co-occurred with immune-related changes beyond the gut. IL-6 is a key mediator in several MIA models, and maternal/fetal IL-6 signaling has been linked to later brain and behavioral outcomes (57, 58). IL-17A has also been implicated in selected MIA models, particularly those connecting maternal Th17 responses with cortical and behavioral alterations in offspring (59, 60). In our study, plasma IL-6 increased in adult MIA offspring, while IL-17 changes were not uniform across groups. This observation may be relevant to SCZ-related neurodevelopmental vulnerability, as elevated circulating IL-6 has been reported in some patient cohorts. However, inflammatory profiles are heterogeneous and cannot be considered diagnostic (61). In the PFC, cytokine changes occurred together with altered Iba1-based microglial measures, suggesting immune-related cortical alterations after MIA. Nevertheless, these data do not demonstrate that gut immunity caused microglial changes. Moreover, microglial morphology alone does not define microglial function, activation state, or synaptic pruning efficiency (62). In neurodevelopmental contexts, CD68 is associated with increased lysosomal content and neuroinflammation (63), while C3 is often discussed in relation to synaptic tagging, complement-mediated synaptic remodeling, and inflammatory microglial states (64, 65). Our data points that in males, microglia exhibited a more phagocytic/reactive phenotype, although in females, microglia showed a complement-associated phenotype, armed with recognition signals (C3), rather than a strongly phagocytic one. Indeed, if interpreted together with CD68 and C3 analyses, our findings can support changes in microglial activation-associated states, but not direct evidence of altered synaptic pruning or neuronal engulfment.

The synaptic marker and behavioral data support the biological relevance of the model. Indeed, changes in PSD95, VGLUT1, gephyrin, and VGAT are consistent with altered excitatory/inhibitory synaptic balance, a process important for neurodevelopmental circuit organization and cortical network function (66–68). However, these protein measurements do not directly assess synaptic transmission. Similarly, reduced spontaneous alternation, altered social preference, and elevated plus maze changes reflect behavioral domains relevant to neurodevelopmental disorders, including SCZ and autism spectrum disorder (3, 38), but they are not disorder-specific diagnostic readouts. Therefore, these findings are best described as MIA-associated behavioral alterations affecting cognitive-, social-, and anxiety/exploration-related domains.

## 5 Conclusions

The integration of microbiota, IEL, and PFC readouts (**Supplementary Table 2**) suggests that the mucosal changes observed after MIA are part of a broader immune network rather than isolated intestinal alterations. In line with this, the association between ileal mucosa-associated SFB and PFC microglial-related readouts across animals with matched measurements points to coordinated changes across gut and brain immune-related compartments. However, this pooled correlation does not establish a direct SFB-to-microglia causal pathway, nor does it determine whether the association is maintained within each sex or treatment condition. Instead, it may reflect broader MIA-associated programming involving the mucosa-associated microbiota, epithelial-immune interface, IEL compartment, and PFC immune state. Because MIA outcomes vary according to strain, immune stimulus, dose and gestational timing, these findings should be interpreted within the context of the present low-dose LPS protocol in BALB/cByJ mice (19, 20). Overall, this study identifies the ileal mucosa-associated microbiota-IEL interface as a prominent site of long-term immune alteration after MIA and highlights IELs and mucosa-associated bacteria as candidate components for future mechanistic studies of microbiota–immune communication in MIA-associated long-term neuroimmune outcomes (**Graphical Abstract**).

## Data Availability

The microbiome sequencing data presented in the study are deposited in the NCBI SRA repository, accession number PRJNA1525017.
