# Supplementary material for: Mucosal microbiota and intraepithelial lymphocyte remodeling link maternal immune activation to neuroimmune outcomes in female and male offspring
Source: Front Immunol. 2026 Sep 18;17:1945751. doi: 10.3389/fimmu.2026.1945751 (PMC13630991; doi:10.3389/fimmu.2026.1945751)
Supplement: Supplementary file 1 [file SupplementaryFile1.docx]

**SUPPLEMENTAL MATERIAL**

**Mucosal microbiota and intraepithelial lymphocyte remodeling link maternal immune activation to neuroimmune outcomes in female and male offspring**

Ana Raquel Pereira-Santos^1,2,3^, Soraia Jesus-Faria^1,2^, Emanuel Candeias^1,2^, Ana Maranha^1,2^, Paulo Rodrigues-Santos^1,2,4,5,6^, Nuno Empadinhas^1,2^, Sandra Morais Cardoso^1,2,4,6*^

^1^CNC – Center for Neuroscience and Cell Biology, University of Coimbra, Coimbra, Portugal

^2^CIBB – Centre for Innovative Biomedicine and Biotechnology, University of Coimbra, Coimbra, Portugal

^3^Ph.D. Programme in Biomedicine and Experimental Biology (PDBEB), Institute for Interdisciplinary Research, University of Coimbra, Coimbra, Portugal,

^4^FMUC -Faculty of Medicine, University of Coimbra, Coimbra, Portugal

^5^Center for Investigation in Environment, Genetics and Oncobiology, University of Coimbra, Coimbra, Portugal

^6^CACC - Clinical and Academic Centre of Coimbra, Coimbra, Portugal

**Table of Contents**

[Key Resources Table	2](#_Toc190266026)

[Figure S1. Body weight and epithelial cytokine measurements.	5](#_Toc190266029)

[Figure S2. Small-intestinal IEL gating strategy and additional ileal cytokines.	6](#_Toc190266030)

[Figure S3. Supplementary blood characterization..	7](#_Toc190266031)

[Figure S4. Microglial and inflammasome pathway in MIA offspring.	8](#_Toc190266032)

[Figure S5. Behavior Supporting data of MIA model.	9](#_Toc190266033)

Table S1. Summary table with the sample size (n) per experimental group and assay.	11

Table S2. Summary of the main MIA-associated findings	14

# Key Resources Table

| **REAGENT or RESOURCE** | **SOURCE** |
| --- | --- |
| **Antibodies – IHC/IF** |  |
| C3 | Abcam, Cat. No. ab11862 |
| Mouse anti-CD11b | BioRad (Cat. No. MCA711GT) |
| CD68 | BioRad, Cat. No. MCA1957 |
| Rabbit anti-Iba1 | FUJIFILM Wako Chemicals (Cat. No. 019-19741) |
| Goat anti-rabbit Alexa Fluor 488 | Life Technologies (Cat. No. A11008) |
| Goat anti-rabbit Alexa Fluor 594 | Life Technologies (Cat. No. A11012) |
| Goat anti-rat Alexa Fluor 594 | Life Technologies (Cat. No. A11007) |
| **Antibodies – Flow Cytometry** |  |
| TCRβ-FITC | Biolegend (Cat. No.109206) |
| TCRδ-PE | Biolegend (Cat.No. 118108) |
| CD8β-PerCP/Cy5 | Biolegend (Cat. No. 126610) |
| CD8α-APC/Cy7 | Biolegend (Cat. No. 344713) |
| CD4-APC | Miltenyi Biotec (Cat.No. 130-121-131) |
| CD45-PerCP | Miltenyi Biotec (Cat. No. 130-102-469) |
| CD3-FITC | Miltenyi Biotec (Cat. No. 130-119-798) |
| CD8a-PE | Miltenyi Biotec (Cat. No. 130-123-781) |
| Live/Death Aqua | ThermoFisher (Cat.No. L34957) |
| **Antibodies – Western Blotting** |  |
| Mouse anti-PSD95 | Abcam (Cat. No. ab2723) |
| Mouse anti-VGLUT1 | NeuroMab (Cat. No. 75-066 clone N28/9) |
| Mouse anti-VGAT | Synaptic systems (Cat. No. 131005) |
| Mouse anti-Gephyrin | Synaptic systems (Cat. No. 147111) |
| Rabbit anti-phospho-STAT3 (pSer727) | Cell Signaling (Cat. No. SAB4504540). |
| Mouse anti-GAPDH | Milipore (Cat. No. MAB374) |
| Mouse anti-Vinculin | Millipore (Cat. No. MAB3574) |
| **Kits** |  |
| NFκB p65 Total SimpleStep ELISA Kit | Abcam (Cat. No. ab176648) |
| Mouse IL-15 ELISA kit | R&D Systems (Cat. No. DY447-05) |
| Mouse IL-23 ELISA kit | R&D Systems (Cat. No. M2300) |
| Mouse IL-22 ELISA kit | R&D Systems (Cat. No. M2200) |
| Mouse IFN-γ ELISA Kit | R&D Systems (Cat. No. MIF00) |
| Mouse IL-6 ELISA Kit | R&D Systems (Cat. No. M6000B) |
| Mouse IL-1β Quantikine ELISA | R&D Systems (Cat. No. MLB00C) |
| Mouse IL-10 Quantikine ELISA | R&D Systems (Cat. No. PM1000B) |
| Mouse IL-17 Quantikine ELISA | R&D Systems (Cat. No. DY421-05) |
| NZY Soil gDNA Isolation kit | Nzytech, Lda (Cat. No. MB21802 |
| **Chemicals** |  |
| Hoechst | Invitrogen (Cat. No. H1399) |
| Histopaque 1083 | Sigma (Cat. No. 10831-100mL) |
| Normal Goat Serum | Abbkine (Cat. No. BMS0050) |
| OCT mounting medium | Carl Roth (Cat. No. KMA-0100-51A) |

# Supplementary Data

**Figure S1. Body weight and epithelial cytokine measurements**. (A) Representation of experimental design. (B) Monitorization of animals’ weight during experimental period (n = 5-10 mice per group). (C) ELISA quantification of IL-15 levels in isolated epithelial cells from small intestine and (D) from large intestine (n = 4-9 mice per group). (E) ELISA quantification of IL-23 levels in isolated epithelial cells from small intestine and (F) from large intestine (n = 4-6 mice per group). *p < 0.05; **p<0.01; ***p<0.001; *Statistical comparisons were conducted between Unt and MIA conditions, separately for females and males using Unpaired t-test. Results are expressed with the mean ± SEM. Related to Figure 1.

**Figure S2. Small-intestinal IEL gating strategy and additional ileal cytokines**. (A) Percentage of lymphocytes in the small intestine. (B) Representative plot of IEL subsets in small intestine. (C) TCR subsets quantified by flow cytometry in small intestine (n = 6-9 mice per group). (D) IL-10 and (E) IFN-γ levels in the ileum of MIA offspring by ELISA (n = 4-8 mice per group). (F) Percentage of lymphocytes in the colon. (G) Representative plot of IEL subsets in the colon. (H) TCR subsets quantified by flow cytometry in the colon (n = 3-5 mice per group). (I) IL-10 and (J) IFN-γ levels in the colon of MIA offspring by ELISA (n = 5-7 mice per group). *p < 0.05, **p < 0.01; *Statistical comparisons were conducted between Unt and MIA conditions, separately for females and males using Unpaired t-test or (H) between all conditions against Unt using one-way ANOVA followed by Tukey's multiple comparisons test. &p<0.05; &Statistical comparisons were analyzed between MIA conditions using one-way ANOVA followed by Tukey's multiple comparisons test. Results are expressed with the mean ± SEM. Related to Figure 2.

Figure S3. **Supplementary blood characterization.** (A) Representative plot of CD4 and CD8 cell population in whole blood. (B) Percentage of lymphocytes and (C) TCR subsets in the whole blood (n = 5-6 mice per group). **p<0.01; *Statistical comparisons were conducted between all conditions using one-way ANOVA followed by Tukey's multiple comparisons test. Results are expressed with the mean ± SEM. Related to Figure 4.

Figure S4. Microglial and inflammasome pathway in MIA offspring. (A) Table presenting the differences in microglia filaments length and intersections between experimental groups in percentage. (B-C) Microglia counts per animal and per area in PFC (n=4-5 animals per group). Assessment of (D) NF-κB and (E) IL-1β levels in prefrontal cortex samples by ELISA (n = 4-5 mice per group). *p<0.05; **p<0.01; *Statistical comparisons were conducted between all conditions against Unt using one-way ANOVA followed by Tukey's multiple comparisons test. Results are expressed with the mean ± SEM.Related to Figure 5 and 6.

Figure S5. Behavior Supporting data of MIA model. (A) Excitatory/inhibitory postsynaptic ratio and (B) excitatory/inhibitory presynaptic ratio in PFC of MIA offspring (n = 3-6 mice per group). (C) Representation of motion trace in MIA animals during Open Field test. (D) Evaluation of social preference by quantifying the percentage of time that animals spent sniffing an empty - E - or an unknown animal - S1 - (n = 4-6 mice per group). (E) Evaluation of the immobility time determined by the Tail suspension test (n = 4-6 mice per group). Quantification of the time spent in (F) close arm or (G) open arm in Elevated Plus Maze test (n = 7-8 mice per group). Quantification of the percentage of entries in (H) open arm or (I) close arm in Elevated Plus Maze test (n = 7-8 mice per group). *p < 0.05; **p < 0.01; *Statistical comparisons were conducted between Unt and MIA conditions, separately for females and males using Unpaired t-test or (D-G) using Kruskal-Wallis test followed by Dunn's multiple comparisons test. (D) Social preference statistical comparisons were conducted between E and S1 conditions, separately for Unt and MIA conditions using Paired t-test. Results are expressed with the mean ± SEM. Related to Figure 6.

**Table S1. Summary table with the sample size (n) per experimental group and assay.**

| Assay | Samples | Dams | | Female offspring | | Male offspring | |
| --- | --- | --- | --- | --- | --- | --- | --- |
|  |  | **Unt** | **MIA** | **Unt** | **MIA** | **Unt** | **MIA** |
| IL-6 | Whole blood GD13.5 | 5 | 6 | n/a | n/a | n/a | n/a |
| IL-17 | Whole blood GD13.5 | 4 | 4 | n/a | n/a | n/a | n/a |
| IL-17 | Whole blood After weaning | 3 | 3 | n/a | n/a | n/a | n/a |
| Peyer’s Patches quantification | Small intestine | n/a | n/a | 6 | 14 | 9 | 9 |
| CD11b intensity | Ileum | n/a | n/a | 4 | 5 | 5 | 7 |
| NF-kB | Ileum | n/a | n/a | 8 | 8 | 8 | 5 |
| IL-1β | Ileum | n/a | n/a | 6 | 6 | 8 | 6 |
| IL-23 | Ileum | n/a | n/a | 3 | 3 | 7 | 5 |
| IL-6 | Ileum | n/a | n/a | 7 | 5 | 5 | 4 |
| CD11b intensity | Colon | n/a | n/a | 4 | 3 | 4 | 3 |
| NF-kB | Colon | n/a | n/a | 6 | 5 | 7 | 6 |
| IL-1β | Colon | n/a | n/a | 6 | 5 | 6 | 6 |
| IL-23 | Colon | n/a | n/a | 5 | 5 | 5 | 6 |
| IL-6 | Colon | n/a | n/a | 3 | 3 | 4 | 4 |
| TCRγδ CD4^-^CD8^-^(%) | Small intestine | n/a | n/a | 6 | 8 | 9 | 8 |
| TCRγδ CD8αα (%) | Small intestine | n/a | n/a | 6 | 7 | 9 | 10 |
| TCRαβ CD8αβ (%) | Small intestine | n/a | n/a | 5 | 7 | 9 | 7 |
| TCRαβ CD8αα (%) | Small intestine | n/a | n/a | 6 | 7 | 8 | 9 |
| TCRαβ CD4 | Small intestine | n/a | n/a | 6 | 8 | 10 | 8 |
| TCRαβ CD4^-^CD8^-^(%) | Small intestine | n/a | n/a | 5 | 6 | 9 | 8 |
| p-STAT3 | Ileum | n/a | n/a | 5 | 6 | 5 | 7 |
| IL-17 | Ileum | n/a | n/a | 7 | 5 | 7 | 5 |
| IL-22 | Ileum | n/a | n/a | 7 | 7 | 7 | 8 |
| TCRγδ CD4^-^CD8^-^(%) | Large intestine | n/a | n/a | 3 | 3 | 3 | 6 |
| TCRγδ CD8αα (%) | Large intestine | n/a | n/a | 3 | 4 | 3 | 5 |
| TCRαβ CD8αβ (%) | Large intestine | n/a | n/a | 3 | 4 | 3 | 4 |
| TCRαβ CD8αα (%) | Large intestine | n/a | n/a | 3 | 3 | 3 | 5 |
| TCRαβ CD4 | Large intestine | n/a | n/a | 3 | 3 | 3 | 4 |
| TCRαβ CD4^-^CD8^-^(%) | Large intestine | n/a | n/a | 3 | 3 | 3 | 5 |
| p-STAT3 | Colon | n/a | n/a | 4 | 4 | 4 | 3 |
| IL-17 | Colon | n/a | n/a | 6 | 5 | 4 | 5 |
| IL-22 | Colon | n/a | n/a | 6 | 5 | 6 | 5 |
| α-diversity | Ileum mucosa-associated | n/a | n/a | 6 | 11 | 4 | 11 |
| Β-diversity | Ileum mucosa-associated | n/a | n/a | 6 | 11 | 4 | 11 |
| Phylum  (relative abundance) | Ileum mucosa-associated | n/a | n/a | 6 | 11 | 4 | 11 |
| Genus  (relative abundance) | Ileum mucosa-associated | n/a | n/a | 6 | 11 | 4 | 11 |
| SFB  (relative abundance) | Ileum mucosa-associated | n/a | n/a | 6 | 11 | 3 | 7 |
| Bacteroides  (relative abundance) | Ileum mucosa-associated | n/a | n/a | 6 | 11 | 4 | 11 |
| Lactobacillus  (relative abundance) | Ileum mucosa-associated | n/a | n/a | 6 | 11 | 4 | 11 |
| CD4 (%) | Whole blood | n/a | n/a | 12 | 7 | 9 | 9 |
| CD8 (%) | Whole blood | n/a | n/a | 12 | 7 | 10 | 10 |
| TCRγδ CD4^-^CD8^-^(%) | Whole Blood | n/a | n/a | 6 | 7 | 9 | 6 |
| TCRγδ CD8αα (%) | Whole Blood | n/a | n/a | 4 | 6 | 3 | 6 |
| TCRαβ CD8αβ (%) | Whole Blood | n/a | n/a | 4 | 6 | 4 | 6 |
| TCRαβ CD8αα (%) | Whole Blood | n/a | n/a | 4 | 6 | 4 | 6 |
| TCRαβ CD4 | Whole Blood | n/a | n/a | 4 | 6 | 4 | 6 |
| TCRαβ CD4^-^CD8^-^(%) | Whole Blood | n/a | n/a | 6 | 7 | 9 | 5 |
| IL-6 | Plasma | n/a | n/a | 4 | 5 | 5 | 3 |
| IL-17 | Plasma | n/a | n/a | 7 | 5 | 5 | 7 |
| Iba1 intensity | PFC | n/a | n/a | 4 | 6 | 4 | 6 |
| Microglia filaments | PFC | n/a | n/a | 4 | 7 | 5 | 4 |
| Sholl analysis | PFC | n/a | n/a | 4 | 7 | 5 | 6 |
| C3 colocalization | PFC | n/a | n/a | 5 | 4 | 5 | 4 |
| CD68 colocalization | PFC | n/a | n/a | 4 | 5 | 5 | 4 |
| Correlation C3 with SFB | PFC/ Ileum mucosa-associated | n/a | n/a | 1 | 2 | 1 | 4 |
| p-STAT3 | PFC | n/a | n/a | 6 | 6 | 5 | 6 |
| IL-17 | PFC | n/a | n/a | 8 | 6 | 7 | 7 |
| IL-22 | PFC | n/a | n/a | 7 | 6 | 6 | 7 |
| PSD95 | PFC | n/a | n/a | 8 | 8 | 6 | 6 |
| VGLUT1 | PFC | n/a | n/a | 8 | 9 | 7 | 8 |
| Gephyrin | PFC | n/a | n/a | 5 | 4 | 6 | 4 |
| VGAT | PFC | n/a | n/a | 7 | 7 | 7 | 7 |
| OpenField test (distance) | n/a | n/a | n/a | 6 | 11 | 5 | 5 |
| OpenField test (velocity) | n/a | n/a | n/a | 6 | 7 | 5 | 5 |
| T-maze test | n/a | n/a | n/a | 5 | 8 | 5 | 9 |
| 3-Chamber Social test | n/a | n/a | n/a | 5 | 6 | 4 | 4 |
| IL-15 | Epithelial cells/Small intestine | n/a | n/a | 6 | 9 | 5 | 5 |
| IL-23 | Epithelial cells/Small intestine | n/a | n/a | 5 | 6 | 4 | 6 |
| IL-15 | Epithelial cells/Large intestine | n/a | n/a | 4 | 4 | 5 | 3 |
| IL-23 | Epithelial cells/Large intestine | n/a | n/a | 4 | 4 | 6 | 4 |
| IL-15 | Duodenum | n/a | n/a | 4 | 5 | 4 | 4 |
| IL-15 | Jejunum | n/a | n/a | 5 | 5 | 4 | 5 |
| IL-15 | Ileum | n/a | n/a | 6 | 6 | 4 | 4 |
| IL-15 | Colon | n/a | n/a | 6 | 6 | 4 | 5 |
| Lymphocytes (%) | Small intestine | n/a | n/a | 6 | 7 | 9 | 8 |
| TCRαβ (%) | Small intestine | n/a | n/a | 6 | 7 | 9 | 9 |
| TCRγδ (%) | Small intestine | n/a | n/a | 6 | 8 | 9 | 9 |
| IL-10 | Ileum | n/a | n/a | 4 | 7 | 6 | 5 |
| IFN-γ | Ileum | n/a | n/a | 6 | 8 | 7 | 8 |
| Lymphocytes (%) | Colon | n/a | n/a | 4 | 4 | 3 | 4 |
| TCRαβ (%) | Colon | n/a | n/a | 3 | 3 | 4 | 5 |
| TCRγδ (%) | Colon | n/a | n/a | 4 | 3 | 3 | 4 |
| IL-10 | Colon | n/a | n/a | 6 | 5 | 6 | 6 |
| IFN-γ | Colon | n/a | n/a | 6 | 7 | 5 | 7 |
| Lymphocytes (%) | Whole blood | n/a | n/a | 6 | 5 | 5 | 6 |
| TCRαβ (%) | Whole blood | n/a | n/a | 5 | 5 | 5 | 5 |
| TCRγδ (%) | Whole blood | n/a | n/a | 6 | 4 | 5 | 4 |
| Microglia Counts | PFC | n/a | n/a | 4 | 4 | 4 | 5 |
| NF-kB | PFC | n/a | n/a | 5 | 5 | 4 | 5 |
| IL-1β | PFC | n/a | n/a | 5 | 5 | 5 | 5 |
| PSD95/Gephyrin ratio | PFC | n/a | n/a | 6 | 3 | 5 | 4 |
| VGLUT1/VGAT ratio | PFC | n/a | n/a | 7 | 6 | 6 | 4 |
| Social preference test | n/a | n/a | n/a | 9 | 10 | 8 | 12 |
| Tail suspension | n/a | n/a | n/a | 4 | 5 | 5 | 6 |
| EPM (close arm) | n/a | n/a | n/a | 7 | 7 | 9 | 8 |
| EPM (open arm) | n/a | n/a | n/a | 7 | 7 | 9 | 8 |
| EPM (entries open arm) | n/a | n/a | n/a | 7 | 7 | 8 | 8 |
| EPM (entries close arm) | n/a | n/a | n/a | 7 | 7 | 8 | 8 |

**Table S2. Summary of the main MIA-associated findings**

| Compartment/readout | Females | Males | Conservative interpretation |
| --- | --- | --- | --- |
| Ileum innate immune markers | Increased CD11b, NF-κB, IL-1β, IL-23 and IL-6 | Increased CD11b, NF-κB, IL-1β and IL-23,  with reduced IL-6 | Region-specific ileal immune activation with sex-dependent IL-6 regulation |
| IELs | Altered TCRγδ CD8αα and selected TCRαβ CD8αα subsets; reduced TCRαβ CD4 IELs | Altered TCRγδ CD8αα and TCRαβ CD8αβ subsets | IEL remodeling emerges as a major novel component of the ileal immune phenotype, although IEL function and cytokine production were not directly assessed |
| Mucosa-associated microbiota | Altered community structure, with SFB and *Lactobacillus* among the prioritized taxa | Taxonomic changes with less pronounced community separation | Mucosa-associated microbiota changes are associated with, but not proven to drive, the ileal immune phenotype |
| Blood | Increased IL-6 and IL-17 | Increased IL-6 and IL-17 | MIA is associated with systemic cytokine changes, including shared IL-6 elevation and a female-biased IL-17 profile |
| PFC  microglia/cytokines | Increased Iba1 intensity and IL-17; increased C3/Iba1 colocalization | Reduced Iba1 intensity/arborization-related measures; increased IL-22; increased C3/Iba1 and CD68/Iba1 colocalization | MIA is associated with sex-dependent PFC immune-related states, including microglial morphological and reactive-marker changes; these readouts do not establish microglial function |
| Behavior/synaptic proteins | Hyperactivity/exploratory behavior,  social/cognitive-related changes and synaptic marker imbalance | Overlapping and sex-dependent behavioral changes with synaptic marker imbalance | MIA is associated with long-term behavioral and synaptic alterations, but these readouts are not diagnostic endpoints |
